# The impact of Highly Active Antiretroviral Therapy (HAART) on the clinical features of HIV - related oral lesions in Nigeria

**DOI:** 10.1186/1742-6405-7-19

**Published:** 2010-06-25

**Authors:** Olaniyi O Taiwo, Zuwaira Hassan

**Affiliations:** 1 Regional Centre for Oral Health Research and Training Initiatives (RCORTI) for Africa, Jos, Nigeria; 2 AIDS Prevention Initiatives for Nigeria (APIN) Project, Jos University Teaching Hospital, Jos, Nigeria

## Abstract

**Background:**

This study aimed to determine the therapeutic effects of highly active anti-retroviral therapy (HAART) on the clinical presentations of HIV related oral lesions (HIV-ROLs) in an adult Nigerian population.

**Methods:**

A 5 month prospective study on HAART naïve HIV positive adults recruited into the HAART program of an AIDS referral centre. HIV-ROLs were diagnosed clinically by the EEC Clearinghouse on oral problems related to HIV infection. Baseline clinical features of HIV-ROLs was documented by clinical photographs using SONY^® ^5.2 M Cybershot digital camera. Post HAART monthly review was conducted using clinical photographs.

**Results:**

A total of 142 patients were seen. Age range was 19 - 75 years. Mean age was 35.6 ± 10.5 (SD). Eighty (56.3%) were females. Prevalence of HIV-ROLs was 43.7%. Oral candidiasis (22.4%) was the most prevalent HIV-ROL. 114 (83.2%) patients had clinical AIDS at presentation (CDC 1993). 89.4% were placed on Tenofovir/Emtricitabine +`Nevirapine, 9.9% on Tenofovir/Emtricitabine + Efavirenz. There was strong decline in the clinical features of oral candidiasis from a month of commencing HAART. Oral hairy leukoplakia was slow in responding to HAART. Parotid gland enlargement, melanotic hyperpigmentation and Kaposi's sarcoma were more persistent and had slower response to HAART. There was no clinical change noticed in linear gingival erythema.

**Conclusion:**

HAART has different clinical effects on HIV related oral lesions depending on the size, duration of treatment and etiology of the lesions. HIV-ROLs of fungal origin have the fastest response to HAART. These lesions alongside immunologic parameters can be used as indicators of success or failure of antiretroviral therapy.

## Background

In recent years, the management of human immunodeficiency virus (HIV) positive individuals has been based on highly active antiretroviral therapy (HAART) comprising a combination of nucleoside analogue reverse transcriptase inhibitors and at least one protease inhibitor and/or one non-nucleoside analogue reverse transcriptase inhibitor [1,2]. HAART induces a marked reduction of viral replication and increases the CD4+ cell count. Since the introduction of HAART in the mid-1990s, it has been accompanied by a reduction in the frequency of many of the secondary events caused by HIV infection, including some oral lesions [3-5]. The sudden profound reduction in viral burden and improvement in cellular immunity achieved with the use of HAART are the most likely influences on the observed reductions [2].

Oral manifestations of HIV infections are sometimes the first sign of the infection and often indicate its progression to AIDS. They have also been considered of value as indicators of success or failure of antiretroviral therapy. It has been reported that HAART has a marked effect on the prevalence and clinical appearance on HIV- related oral lesions [6]. These effects vary from review of the literatures.

Since the advent of HAART, studies had shown a decline in the prevalence of oral lesions associated with HIV/AIDS. These lesions include: oral candidiasis, hairy leukoplakia, Kaposi's sarcoma, herpes simplex labiali, and periodontal disease [1,2,4-9]. Other studies had reported no change in the prevalence of some of the HIV related oral lesions such as aphthous ulcers [5], salivary gland disease [2], human papillomavirus-associated oral lesions [1,2] and herpes simplex infection [10]. Oral Warts is one lesion that had been reported to have a six-fold "striking increase" with HAART [5]. Complete resolution was reported for a case of Kaposi's sarcoma in a 52 years old homosexual male with a primary HIV infection after being on HAART for 4 months [11]. Nearly all the reported studies had been conducted in industrialized countries and literatures concerning the behavior of HIV related oral lesions in patients undergoing HAART is scarce [12]. This study therefore aimed to determine the therapeutic effects of HAART on the clinical presentations of HIV related oral lesions in an adult Nigerian population.

## Materials and methods

The study took place at the adult wing of the AIDS Prevention Initiative for Nigeria (APIN) centre, Jos, Nigeria. This is a referral center specialized in the diagnosis and management of HIV infection. Patients examined were those who have been confirmed to be HIV positive through western blot and/or the use of double ELISA. These patients were those recruited into the HAART program of the centre. The study protocol was approved by the ethical committee of the Jos University Teaching Hospital and each patient gave written informed consent.

Inclusion criteria established that patients were HAART naïve. Oral lesions were diagnosed clinically according to the criteria established by the European Economic Community Clearinghouse on oral problems related to HIV infection [9]. Oral examinations were performed by a Dental surgeon trained in the identification of HIV related oral lesions. Where multiple lesions were seen (in the same patient) at the time of clinical evaluation, each lesion was considered independently for the analysis.

The baseline clinical status of soft oral tissues of all the included patients was documented on their first clinical appearance. The location, clinical characteristics and date lesion was seen were documented by clinical photographs. This was by the use of a SONY^® ^5.2 M Cybershot digital camera. Pictures were transferred into coded folders (for each patient) created in a Toshiba Satellite laptop. The patients were monitored monthly (for 5 months) at the clinic when they come for their monthly HAART medications and the same procedure was repeated. No antifungal therapy was administered post diagnosis of oral lesions.

For each patient, the following information was also recorded: age, sex, smoking and alcohol use, clinical stage of infection (using the clinical categories of the1993 revised CDC classification system for HIV infection), type of HAART prescribed and the HIV related oral lesion(s) noticed. Also documented were the CD4 cells (count/ml) and the HIV RNA copies/ml at baseline, 12 and 24 weeks after.

The effects of HAART on the lesions were recorded as a decrease in the size of the lesion, resolution, no change and the appearance of a new lesion (or recurrence). Effects on the immunologic parameters were based on the relative changes in the different categories. CD4 cells counts ≥500/ml was classified as "marginally immunodeficient", CD4 cell count of >200 to <500/ml as "mildly immunodeficient" and CD4 cell count of ≤200/ml as "severely immunodeficient". Grade 1 classification for viral load was 'undetectable' to ≤10,000 copies/ml while Grade 2 was >10,000 copies/ml. Statistical analysis which includes cross-tabulations, frequencies and Chi - Square test was done using SPSS software version 15.0.

## Results

A total of 142 patients were seen. Age range was 19 - 75 years. Mean age was 35.6 ± 10.5 (SD). 80 (56.3%) were females. At the time of the clinical examinations, 73.2% of the patients said they were non drinkers, 18.3% drink alcoholic beverages occasionally. Also, as regards tobacco use, 93% of the patients were non smokers, 5.6% had stopped smoking while only 2 (1.4%) were active smokers. A total of 62 (43.7%) patients had at least an oral manifestation of HIV/AIDS. 22 (35.5%) of this number (62) had multiple lesions with the maximum number noticed per patient being 4.

According to the 1993 revised CDC classification system for HIV infection, 114 (83.2%) patients had clinical AIDS at presentation (Table 1). The immunologic status at presentation showed an average CD4 count (cells/ml) of 148.5 ± 117.5 (SD) and a range of 553. The average viral load (copies/ml) was 163,831.9 ± 279,964.9 (SD) and a range of 1,664,700. At the moment of their first oral examinations, only one patient was classified as "marginally immunodeficient" and 21.1% were in the viral load group - Grade 1.

**Table 1:** Disease Stage for 142 HIV infected Nigerian patients using the 1993 Revised CDC Classification System for HIV Infection

CD4+ T cell categories (cells/mm^**3**^)	Clinical categories		
	
	A	B	C
	Asymptomatic, acute (primary) HIV	Symptomatic, not A or C Conditions	AIDS Indicator Conditions
≥500	A1 (1)	B1 (0)	C1 (0)
200-499	A2 (10)	B2 (12)	C2 (16)
<200	A3 (19)	B3 (35)	C3 (44)

Persons in categories A3, B3, C1, C2, and C3 have **AIDS **under the 1993 surveillance case definition i.e. 19 + 35 + 44 + 16 = 114. This gives **83.2% **for the proportion with AIDS (computed with 137 as the total number due to 5 missing CD4 counts)

Candidosis was the most prevalent oral manifestations of HIV/AIDS (22.4%). It had equal presentations in both gender. Erythemathous candidiasis (EC, 10.6%) was the most common clinical type noticed. Melanotic hyperpigmentation (MH) was the next most common presentation (18.3%) followed by oral hairy leukoplakia (9.9%) (Table 2). Nearly all the patients (89.4%) were placed on two NRTIs (Tenofovir/Emtricitabine) plus an NNRTI (Nevirapine). 14 (9.9%) patients were placed on Tenofovir/Emtricitabine but with a separate NNRTI (Efavirenz). Only a patient was on the combination of (Lamivudine/Zidovudine) and a protease inhibitor (Indinivir). Table 3 shows the effects of HAART on the different immunologic classifications of the patients from 'start' to 24 weeks.

**Table 2:** Prevalence of oral manifestations of HIV/AIDS in 142 HIV infected Nigerian patients.

Type	Male		Female		Total	
						
Any oral disease						
**Candidal lesion**	*N*	%	*n*	%	N	%
Pseudomembraneous Candidiasis	4	2.8	5	3.5	9	6.3
Angular Cheilitis	3	2.1	5	3.5	8	5.8
Erythematous Candidiasis	9	6.3	6	4.2	15	10.6
**Total**	**16**	**11.2**	**16**	**11.2**	**32**	**22.4**
Linear Gingival Erythema	1	0.7	-	-	1	0.7
Oral Hairy Leukoplakia	6	4.2	8	5.6	14	9.9
Herpes Simplex	1	0.7	-	-	1	0.7
Melanotic Hyperpigmentation	12	8.5	14	9.9	26	18.3
Oral ulcerations	4	2.8	1	0.7	5	3.5
Kaposi's Sarcoma	3	2.1	2	1.4	5	3.5
Enlarged salivary gland	3	2.1	3	2.1	6	4.2
Xerostomia	5	3.5	1	0.7	6	4.2
**Presence of HIV-related oral lesions**	**30**	**21.1**	**32**	**22.5**	**62**	**43.7**

**Table 3:** Effects of HAART on the immunologic status of HIV infected Nigerian patients

	CD4 cells counts (cells/ml)			Viral loads (copies/ml)		
**No of patients**	**% of those with (≥500)**	**% of those with (200 - 499)**	**% of those with (<200)**	**No of patients**	**% of those in Grade 1 ≤10,000**	**% of those in Grade 2 >10,000**

**Start **N = 137	0.7	27.7	71.6	N = 142	21.1	78.9
**12 weeks **N = 82	6.1	50	43.9	N = 97	84.5	15.5
**24 weeks **N = 97	6.2	51.5	42.3	N = 85	84.7	15.3

### Effects of HAART on HIV-related oral lesions

**Figure 1:**
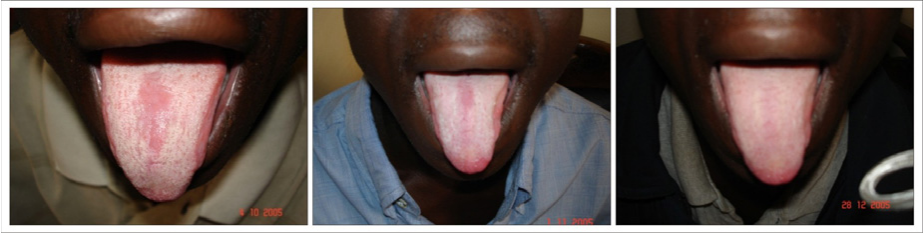
**Erythemathous Candidiasis **Patient on: Tenofovir/Emtricitabine + Nevirapine

**Figure 2:**
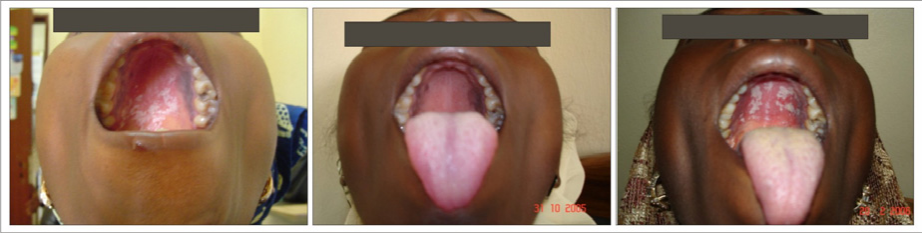
**Pseudomembraneous Candidiasis (Re-occurrence) **Patient on: Tenofovir/Emtricitabine + Nevirapine

Depending on the extent of the lesions at presentation, all the available cases of pseudomembraneous candidiasis, angular cheilitis, erythematous candidiasis (figure 1) and oral ulcers had disappeared by the third month of observation. The response was a strong decrease in candidiasis most especially pseudomembraneous candidiasis whose resolution had been noticed starting a month after commencing HAART. A new case (recurrence) of pseudomembraneous candidiasis was noticed 4 months after the lesion had disappeared (figure 2).

**Figure 3:**
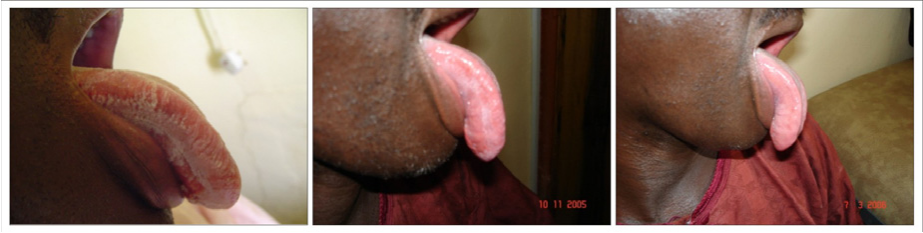
**Oral Hairy Leukoplakia **Patient on: Tenofovir/Emtricitabine + Nevirapine

Oral hairy leukoplakia (figure 3) was slow in responding to HAART. There was no significant clinical change on some of the lesions in the first month of using HAART. A gradual decrease in the size of the lesion was observed for months with eventual disappearance of some by the end of 5 months.

**Figure 4:**
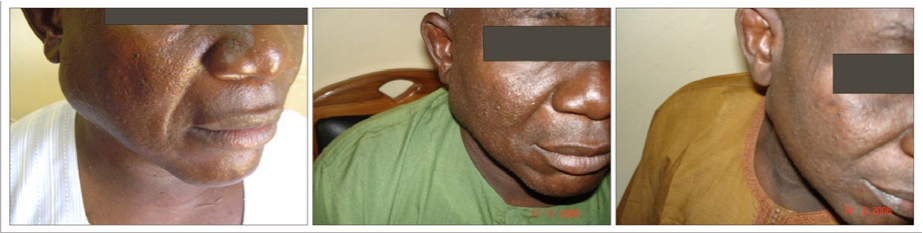
**Parotid Gland Enlargement **Patient on: Tenofovir/Emtricitabine + Efavirenz

**Figure 5:**
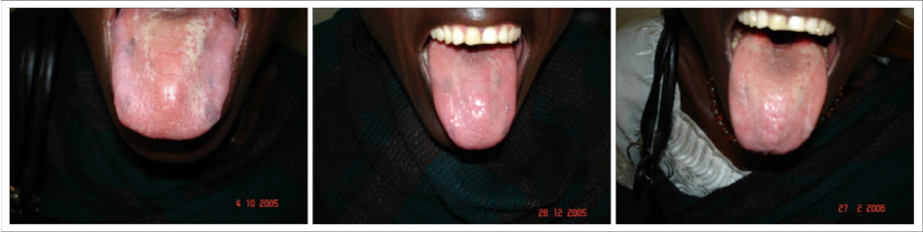
**Melanotic Hyperpigmentation **Patient on: Tenofovir/Emtricitabine + Efavirenz

**Figure 6:**
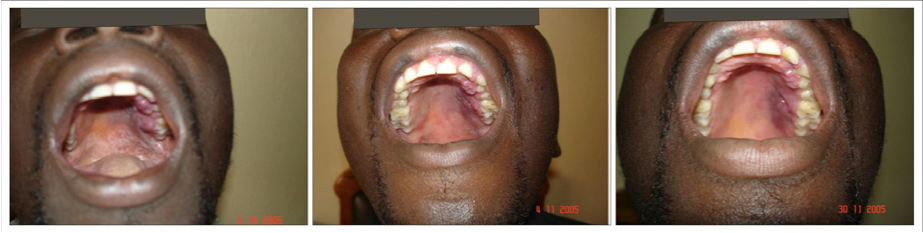
**Kaposi's Sarcoma **Patient on: Tenofovir/Emtricitabine + Nevirapine

Parotid gland enlargement (figure 4), melanotic hyperpigmentation (figure 5) and Kaposi's sarcoma (figure 6) were more persistent and had slower response to HAART. For all the lesions monitored, none was able to achieve complete resolution during the period of observation. For the first few months, there was negligible change in the clinical features of melanotic hyperpigmentation. Gradual decrease in size was also noticed for parotid gland enlargement and Kaposi's sarcoma. There was no change observed in the only case of linear gingival erythema noticed throughout the duration of the study. Table 4 shows the relationship between the prevalence of the oral manifestations of HIV/AIDS and the immunologic parameters from the commencement of HAART to 24 weeks.

**Table 4:** Prevalence of Oral manifestations of HIV/AIDS in relation to immunologic status

Immunologic parameter	week	Mean Value ± SD	Total number of patients	No with HIV/AIDS oral lesions	Prevalence of oral manifestations of HIV/AIDS (%)	p-value
**CD4 counts (cells/ml)**	0	148.45 ± 117	142*	62	43.7	0.000
	12	245.28 ± 144	82	12	14.6	
	24	253.87 ± 147	97	14	14.4	
**Viral load (copies/ml)**	0	163,831.91 ± 279,964	142	62	43.7	0.000
	12	41,122.27 ± 151,724	97	14	14.4	
	24	13,900.28 ± 52,352	85	12	14.1	

*A total of 142 patients was seen at start but only the CD4 counts of 135 patients was available

## Discussions

It is well known that HIV-positive patients with CD4+ lymphocyte counts less than 200 cells/ml are severely immunocompromised and the HIV-positive patient with viral load greater than 10,000 copies/ml show active viremia [12]. In both Cases, these values are indicators of progression to AIDS. These markers are used to indicate success or failure of antiretroviral therapy. Oral manifestations of HIV infections which are sometimes the first sign of the infection can also indicate its progression to AIDS. This is because these lesions appear as a result of declining immune competence. They have also been considered of value as indicators of success or failure of antiretroviral therapy.

With 114 (83.2%) of our patients having frank AIDS at baseline (CDC 1993), a prevalence of 43.7% was noticed for HIV related oral lesions. There was an appreciable immunologic reconstitution in the patients observed at 12 and 24 weeks compared to baseline (Table 3). This we believe may be responsible for the positive changes noticed in the clinical features of the HIV-ROLs studied. During the period of study, HIV-ROLs of fungal origin (pseudomembraneous, erythemathous candidiasis and angular cheilitis) experienced significant changes - complete resolution. This change was gradual and became noticeable by the end of the first month of using HAART. The rate of resolution also depended on the size of the lesion at presentation. It has been proposed that this could be as a result of the effects of HAART on secreted aspartyl proteinases (SAPs) which are an important virulence factor of *candida*. This is apart from the improvement in the immunological response of the host from HAART [13]. Our findings demonstrating a strong decline in the clinical features of oral candidiasis agrees with previous reports.

The re-occurrence of a new lesion of pseudomembraneous candidiasis 4 months after the resolution of the same in a female patient may be an evidence of immune reconstitution inflammatory syndrome (IRIS). 'An IRIS event is defined as either a first presentation or a paradoxical worsening of a pre-existing condition after the initiation of HAART in the presence of rising CD4 counts and falling HIV-1 RNA levels, if measured'[14]. The CD4 counts (cells/ml) of this patient rose from 73 to 269 while the viral load (copies/ml) dropped from 514,298 to 288. Also, oral candidiasis had been shown to be one of the lesions evident of IRIS in patients on HAART [14]. Oral hairy leukoplakia which is of viral origin was slower in its response to HAART. The effect on OHL may be due to the direct effect of HAART on the lesion. It may also be due to the host defense mechanism against opportunistic organisms such as EBV, the etiologic agent of OHL as a result of the gradual restoration of CD4+ cells [15]. Clinical features of HIV-ROLs of neoplastic and auto-immune origin such as oral Kaposi's sarcoma, melanotic hyperpigmentation, and salivary gland enlargement were more protracted in their response to HAART. None of them achieved complete resolution during the study period. This might be due to the size of the lesions seen and duration of treatment (as in this study). A study had reported a complete resolution of oral Kaposi's sarcoma within 4 months of antiretroviral therapy [11]. This achievement might be as a result of the size of the lesion. Very few studies had reported the effects of HAART on melanotic hyperpigmentation basically due to its low prevalence in their areas. A study actually reported an increase in the prevalence of melanotic hyperpigmentation in their patients on HAART. This was linked with increase melanin production in the epithelium associated with increased release of a melanin stimulating hormone (a-MSH) as a result of systemic ketoconazole and zidovudine therapy. Only a patient in our cohort had a combination of HAART which included zidovudine. Salivary gland enlargement had been shown in other studies to actually increase with the advent of HAART [5] while others had reported no change of the same during treatment with HAART [2,16]. These conflicting findings may be due to the use of different HAART regimen and different treatment durations.

The reduction in the prevalence of HIV related oral lesions in our study from week 0 to 12 was very significant. A Greek study on the effects of PI - HAART on the prevalence of oral lesions in HIV -1 infected patients also reported similar findings [17]. However, the prevalence over the next 12 weeks (to week 24 in our study) was very minimal. This could be as a result of the lesions of viral, auto-immune and neoplastic origin which we observed had a protracted response to HAART.

There were some limitations to this study. It was assumed that the patients adhered strictly to the prescriptions given since for the duration of the study, we only had a contact with them once a month. Also, we could not rule out the possibility of concomitant drug use as self medication is rife in many parts of the country. Some of these drugs could also have effects on the HIV-ROLs. As the study progressed, there was gradual attrition of the patients. This was due to many factors which includes the demise of some of the patients, inability to make appointment dates due to lack of funds to travel, misinterpretation of appointment dates and medical complications limiting movement. Nevertheless, our study was able to show the varied effects of HAART on HIV-ROLs in an adult Nigerian population. The observed effects were contingent on the size of the lesion, duration of treatment and the etiology of the lesions. These lesions alongside immunologic parameters can be used to monitor the responses of patients to HAART.

## Competing interests

The authors declare that they have no competing interests.

## Authors' contributions

OOT conceived the idea, designed the study and wrote the protocol. ZH did the overall supervision, patient coordination and follow - up. OOT prepared the recording leaflets, took the digital pictures, entered them in to the computer and performed the analysis. Both OOT and ZH participated in data collection and writing of the manuscript. All authors have read and approved the final manuscript.
